# Binding of hnRNP H and U2AF65 to Respective G-codes and a Poly-Uridine Tract Collaborate in the N50-5'ss Selection of the REST N Exon in H69 Cells

**DOI:** 10.1371/journal.pone.0040315

**Published:** 2012-07-05

**Authors:** Carlos Ortuño-Pineda, José Manuel Galindo-Rosales, José Victor Calderón-Salinas, Nicolás Villegas-Sepúlveda, Odila Saucedo-Cárdenas, Mónica De Nova-Ocampo, Jesús Valdés

**Affiliations:** 1 Departamento de Bioquímica, Centro de Investigación y de Estudios Avanzados del I.P.N., México D.F., México; 2 D1epartamento de Biomedicina Molecular, Centro de Investigación y de Estudios Avanzados del I.P.N., México D.F., México; 3 Departamento de Histología, Facultad de Medicina, Universidad Autónoma de Nuevo Léon, Monterrey N.L. México; 4 División de Genética, Centro de Investigación Biomédica del Noreste, Instituto Mexicano del Seguro Social, Monterrey N.L., México; 5 Programa Institucional de Biomedicina Molecular, Escuela Nacional de Medicina y Homeopatía-IPN, México D.F., México; International Centre for Genetic Engineering and Biotechnology, Italy

## Abstract

The splicing of the N exon in the pre-mRNA coding for the RE1-silencing transcription factor (REST) results in a truncated protein that modifies the expression pattern of some of its target genes. A weak 3'ss, three alternative 5'ss (N4-, N50-, and N62-5'ss) and a variety of putative target sites for splicing regulatory proteins are found around the N exon; two GGGG codes (G2-G3) and a poly-Uridine tract (N-PU) are found in front of the N50-5'ss. In this work we analyzed some of the regulatory factors and elements involved in the preferred selection of the N50-5'ss (N50 activation) in the small cell lung cancer cell line H69. Wild type and mutant N exon/β-globin minigenes recapitulated N50 exon splicing in H69 cells, and showed that the N-PU and the G2-G3 elements are required for N50 exon splicing. Biochemical and knockdown experiments identified these elements as U2AF65 and hnRNP H targets, respectively, and that they are also required for N50 exon activation. Compared to normal MRC5 cells, and in keeping with N50 exon activation, U2AF65, hnRNP H and other splicing factors were highly expressed in H69 cells. CLIP experiments revealed that hnRNP H RNA-binding occurs first and is a prerequisite for U2AF65 RNA binding, and EMSA and CLIP experiments suggest that U2AF65-RNA recognition displaces hnRNP H and helps to recruit other splicing factors (at least U1 70K) to the N50-5'ss. Our results evidenced novel hnRNP H and U2AF65 functions: respectively, U2AF65-recruiting to a 5'ss in humans and the hnRNP H-displacing function from two juxtaposed GGGG codes.

## Introduction

The alternative splicing mechanism generates vast numbers of protein isoforms amplifying the eukaryotic proteome and affecting human genetic diseases. Frequently, such diseases are caused by mutations in splicing regulatory sequences [1] or by changes in the relative concentrations of splicing factors affecting the spliceosomal composition [2].

**Figure 1:**
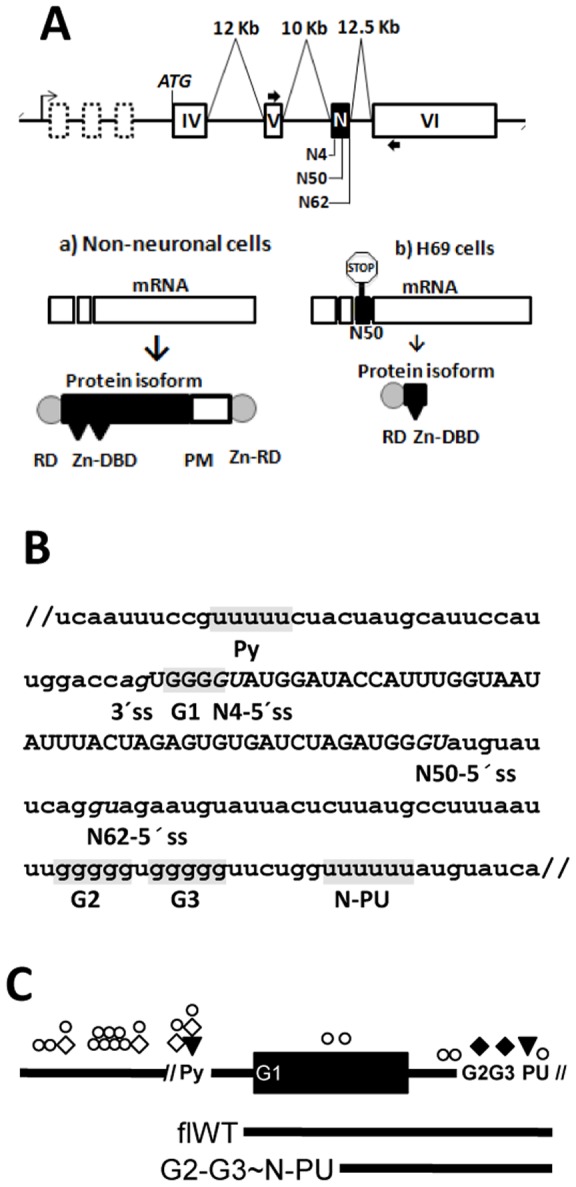
Splicing regulatory elements around the N exon. (**A**) Schematic representation of the REST gene (drawing not to scale) showing the transcription start site (right arrow), introns (thin lines), lengths of relevant introns, constitutive exons coding for the UTR (dashed boxes) and for the REST protein (white boxes), including the alternative N exon (black box); the relative positions of the alternative N4-, N50- and N62-5'ss appear underneath. Thick arrows indicate the positions of the primers used to amplify the mRNA variants by RT-PCR. The main possible mRNAs and proteins expressed in **a**) non-neuronal cells and **b**) SCLC H69 cells are depicted also. Splicing of the N50 exon introduces a stop codon. Nomenclature of protein domains: RD, repressor domain; Zn-DBD, zinc-finger DNA-binding domain; PM, Proline-rich motif; and Zn-RD, zinc-finger repressor domain. (**B**) Genomic DNA from H69 and MRC5 cells was used to amplify 163 bp (between virgules) that include the N exon by PCR and the sequence identity between the cell lines was confirmed. The names of the relevant transcript elements are shown under the sequence. The AG-UG dinucleotides, corresponding to the 3′ss, and the N4-, N50- and N62-5'ss appear in italics; the upstream Py, G1-G3, and N-PU elements are boxed in gray. (**C**) Unscaled drawings of the RNA molecules used in this work. The top one is coded by the minigene constructs (271 nt upstream, 62 nt of the N exon, and 72 nt downstream); the sequences between virgules correspond to those shown in B. The full-length wild type (flWT) and G2-G3∼N-PU probes are also shown. Mutant versions (without both elements or without N-PU) of the G2-G3∼N-PU probe were made also. The putative RNA binding sites for U2AF65 (triangles), hnRNP H (black diamonds), TIA-1/TIAR (open circles), and nSR100 (white diamonds) were predicted using the Splicing Rainbow software; the symbols indicate their relative positions in the RNA molecules.

A clear example of splicing pattern-induced gene expression changes is the neural phenotype observed in small cell lung cancer (SCLC). This is partly due to the expression of the truncated (t) REST (RE1-Silencing Transcription Factor) isoform (Fig. 1A, b) [3], [4], which modifies the expression of neuron-restricted genes otherwise silenced by REST in non-neuronal cells. In several SCLC cell lines, tREST is coded by the REST-N50 (a.k.a. sNRSF) mRNA variant, which carries a premature stop codon due to the neuronal (N) exon inclusion [4]. The N exon is localized between exons V and VI, and its variable length of 4, 50 or 62 nucleotides (nt) is determined by the selection of one of three alternative 5'ss (here named N4-, N50- and N62-5'ss, respectively)(Fig. 1A)[5], [6]. Whereas in neuroblastomas the N4-5'ss is mainly selected [6], in the SCLC H69 cell line the N50-5'ss selection has been observed only [4].

Early during spliceosomal assembly, the 3'ss is recognized by the small subunit of the essential splicing factor U2AF, U2AF35. The large subunit, U2AF65, interacts with U2AF35 and binds to polypyrimidine tracts (Py) upstream of the 3'ss, by forming unique hydrogen bonds with the Uracil edges and promoting the U2 snRNP binding to the branch point site [7], [8]. A systematic analysis of constitutive and alternative exons, revealed an enrichment of polyuridine tracts (PU) within 100 nt following their 5'ss which promote the inclusion of the adjacent exons [9] by TIA-1/TIAR-mediated [10] or U2AF65-mediated [11] U1 snRNP 70K recruitment to the 5'ss. Also, neural-specific exons are activated by nSR100 (neural-specific Ser/Arg repeat-related protein of 100 kDa), which binds to proximal (1000 nt around the exon) intronic C/U-rich motifs [12].

Recently, an array composed of G-rich [13], have been implicated in the coordinated splicing regulation of neural-specific alternative exons. These events involve other splicing signals and factors as well. For example, the glutamate receptor NMDA R1 neuronal exon cassette C1 is combinatorially regulated by an exonic UAGG element and a GGGG code proximal to the 5'ss [14], and the function of these elements is linked to the splicing factor hnRNP H. The splicing of the *c-src* N1 exon is regulated by the cooperative binding of hnRNP H and hnRNP F to an intronic splicing enhancer, localized downstream of the 5'ss in front of the exon [15]. The alternative splicing of several exons of the (PLP)/DM20 gene require hnRNP H and hnRNP F recognition of a complex GGGG-codes array [16], and the insulin exon 11 is activated when hnRNP F binds intronic UAGGGA elements in front of the upstream exon 10 and functionally interacts with SRSF1 (SF2/ASF) bound to exon 11 [17].

A sequence analysis of the N exon environment (Fig. 1B) revealed a PU, here referred as N-PU, localized 60 nt downstream of the N50-5'ss, and three GGGG codes that may participate in its activation: one comprising the N4 and juxtaposed to the N4-5'ss (G1) and two downstream (G2-G3) the N exon.

Here we investigated N50 exon activation in H69 cells. N exon/β-globin minigenes recapitulated N50 exon splicing when transfected into H69 cells, and mutant minigenes showed that the N-PU and the G2-G3 elements are required for N50 exon splicing. Biochemical and knockdown experiments showed that these elements are targets for U2AF65 and hnRNP H, respectively, and that they are also required for N50 exon activation. In agreement with the unique N50-5'ss selection in H69 cells and the absence of N exon activation in the proxy to normal MRC5 cells, U2AF65, hnRNP H and other splicing factors were highly expressed in H69 cells. CLIP experiments revealed that hnRNP H RNA-binding occurs first and is a prerequisite for U2AF65 RNA binding and EMSA experiments showed that binding of U2AF65 to the N-PU displaces hnRNP H from the G2-G3 codes; these hnRNP H and U2AF65 functions have not been previously described. Finally, U2AF65 appears to promote U1 snRNP 70K recruiting for N50-5'ss activation. Altogether, these findings are in agreement with the model in which in MRC5 cells the N exon could be repressed by hnRNP H binding to the GGGG codes, whereas in H69 cancer cells, such repression might be relieved by U2AF65 binding to the N-PU, whose recruiting appears to be facilitated by hnRNP H, leading to N exon activation.

## Results

### The N-PU, G2-G3 codes, U2AF65, hnRNP H are essential elements and factors for N50 activation and tREST expression

**Figure 2:**
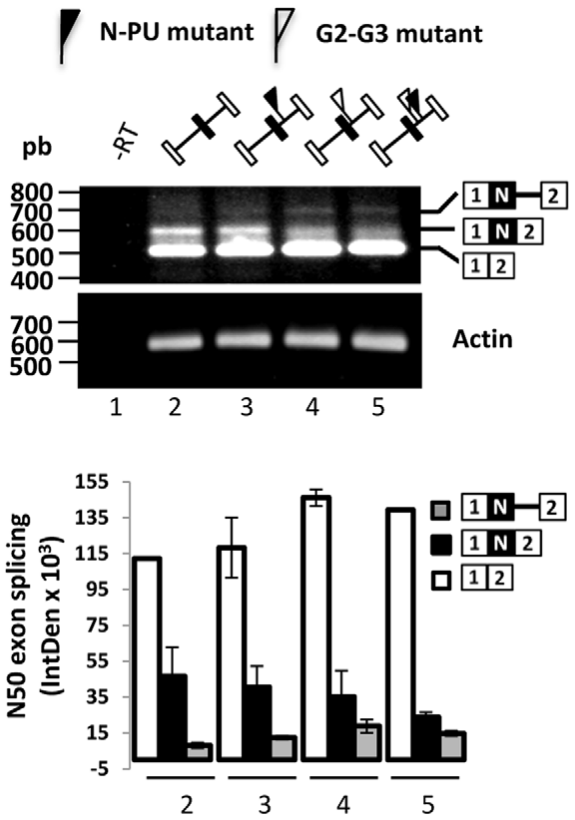
N-PU and G2-G3 codes are required for N50 exon activation in H69 cells. Mutant N-PU and G2-G3 elements negatively affect N50 exon inclusion. Cells were transfected with the wild type (lanes 1 and 2) or mutant N50/β-globin minigenes (lanes 3–5). 24 h post transfection, N exon inclusion between β-globin exons 1 and 2 was monitored by RT-PCR (with primers T7 and SP6). No RT (- RT) PCR control reaction appears in lane 1. The plot below shows the quantification of the spliced and partially spliced mRNAs in the indicated lanes. The absence of either element decreased N50 exon activation, albeit the lack of the G2-G3 rendered more exon skipping and partially spliced products.

In H69 cells, the splicing of the N50 exon in the REST pre-mRNA originates the REST-N50 mRNA variant and its corresponding truncated protein [4], however the mechanisms and signals involved in such splicing mechanism are less understood. To analyze the cues involved in the N50-5'ss activation in H69 cells, a minigene construct containing N50 exon sequences (Fig. 1C) cloned between exons 1 and 2 of the β-globin cDNA was made, transfected into H69 cells, and the in vivo splicing products were analyzed by RT-PCR using primers targeted to the β-globin exons (for primers and PCR conditions, see Fig. S1). The N exon/β-globin minigene recapitulated the splicing pattern of the N50 exon (Fig. 2, lane 2).

Around the N exon, multiple putative targets of splicing factors were identified (Fig. 1C). A N-PU (downstream of the N62-5'ss), probably recognized by the splicing activator U2AF65 which has been reported to promote U1 snRNP recruitment to weak 5'ss [11]. Also, several PU sites were found; these are cognate targets of the activator of the neurofibromatosis type 1 exon 23a TIA-1/TIAR [18]. Three GGGG codes (the N4 conforming code G1, juxtaposed to the N4-5'ss, and codes G2 and G3 localized 6 nucleotides upstream the N-PU) were found. These elements may be recognized by the splicing modulator of neuronal exons hnRNP H [14]. Finally, four C/U elements were identified, which could be probably recognized by the activator of the REST N4 exon, nSR100 [19].

This complex array of elements suggest their possible involvement in the preferred N50- over N4- and N62-5'ss selection in H69 cells. To test this, N-PU mutant (UUUUUU to GAUAUC), G2-G3 mutant (GGGGGUGGGGG to CACGGUCACGG) and G2-G3∼N-PU double mutant minigenes were analyzed as before. We found that the single mutants rendered 20–30% less N50 exon inclusion, coordinated with a proportional increase in N exon-skipped and partially spliced products (Fig. 2, lanes 3 and 4). In the double mutant N50 splicing was further reduced (Fig. 2, lane 5). These results suggest that both N-PU and G2-G3 elements are required for N50 exon activation.

**Figure 3:**
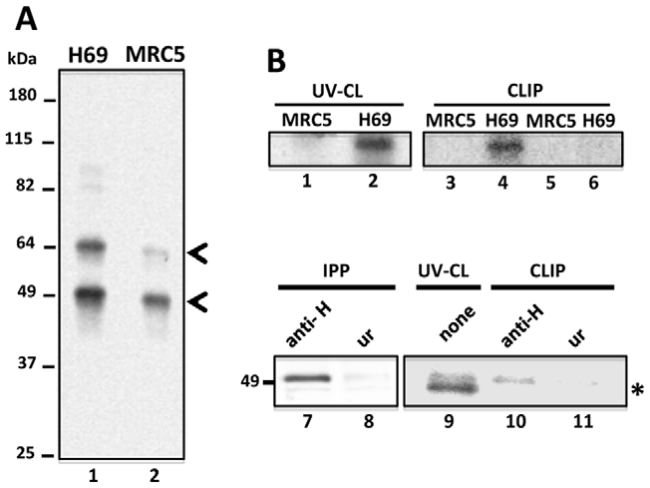
U2AF65 and hnRNP H bind to probes containing the N-PU and the G2-G3 codes. (**A**) flWT N exon synthetic transcripts containing the three GGGG codes and the N-PU, and NEx from H69 (lane 1) and MRC5 (lane 2) cells were used in UV crosslinking experiments. Enriched p64 and p50 proteins in H69 are indicated with arrowheads. (**B**) UV crosslinking (lanes 1 and 2) and CLIP (lanes 3–6) assays were carried out with G2-G3∼N-PU synthetic RNAs, NEx from MRC5 (lanes 1, 3 and 5) or H69 cells (lanes 2, 4 and 6), and with antibodies against U2AF65 (lanes 3, and 4) or an unrelated antigen (lanes 5 and 6). Immunoprecipitation (lanes 7 and 8), UV crosslinking (lane 9) and CLIP (lanes 10 and 11) assays were carried out with the same synthetic RNAs and NEx from H69 cells, using antibodies against hnRNP H (lanes 7 and 10, respectively) or an unrelated antigen (lanes 8 and 11, respectively). A low molecular mass unidentified protein cross-reacted with the probe (asterisk).

Given that there are no N exon sequence differences between H69 and MRC5 cell lines (not shown), N50 activation in H69 cells probably obeys to cellular context. Therefore, we investigated whether the nuclear factors U2AF65 and hnRNP H from H69 cells could differentially recognize the N exon RNA. Nuclear extracts (NEx) from H69 and MRC5 cells were UV cross-linked to synthetic flWT transcripts (Fig. 1C). Albeit enhanced signals of p100 and p90 were observed in H69 nuclear proteins, the signals of p65 and p49 were particularly strong (Fig. 3A, lanes 1 and 2), which might correspond to U2AF65 and hnRNP H, respectively. To confirm the identity of these proteins and to further delineate the elements recognized by these factors, NEx of both cell lines and a shorter version of the probe (G2-G3∼N-PU) were used in CLIP assays. As before, a 65-kDa nuclear protein from H69 cells was UV crosslinked to the probe (Fig. 3B, compare lanes 1 and 2), and U2AF65 was precipitated from H69 (lane 4) but not from MRC5 NEx (lane 5). Unrelated antibodies rendered no CLIP signals with either cell line (lanes 5 and 6). In agreement with this, complex formation between rU2AF65 and N-PU mutant probes is reduced by 80% in EMSA (Fig. S2), suggesting that the N-PU is necessary for U2AF65 recruiting to the N exon alternative 5'ss. Likewise, hnRNP H was precipitated only from H69 cells after UV crosslinking to the G2-G3∼N-PU probe, whereas no signal was observed when an unrelated antibody was used (Fig. 3B, lanes 10 and 11, respectively). HnRNP H was detected in immunoprecipitation-only and UV crosslinking-only control reactions (Fig. 3B, lanes 7 and 9, respectively); the UV crosslinking reactions showed a 48-kDa protein, probably corresponding to TIA-1/TIAR, and no signal was observed with an unrelated antibody immunoprecipitation (lane 8). Collectively, these results suggest that U2AF65 and hnRNP H bind the N-PU and the G2-G3 codes, respectively.

**Figure 4:**
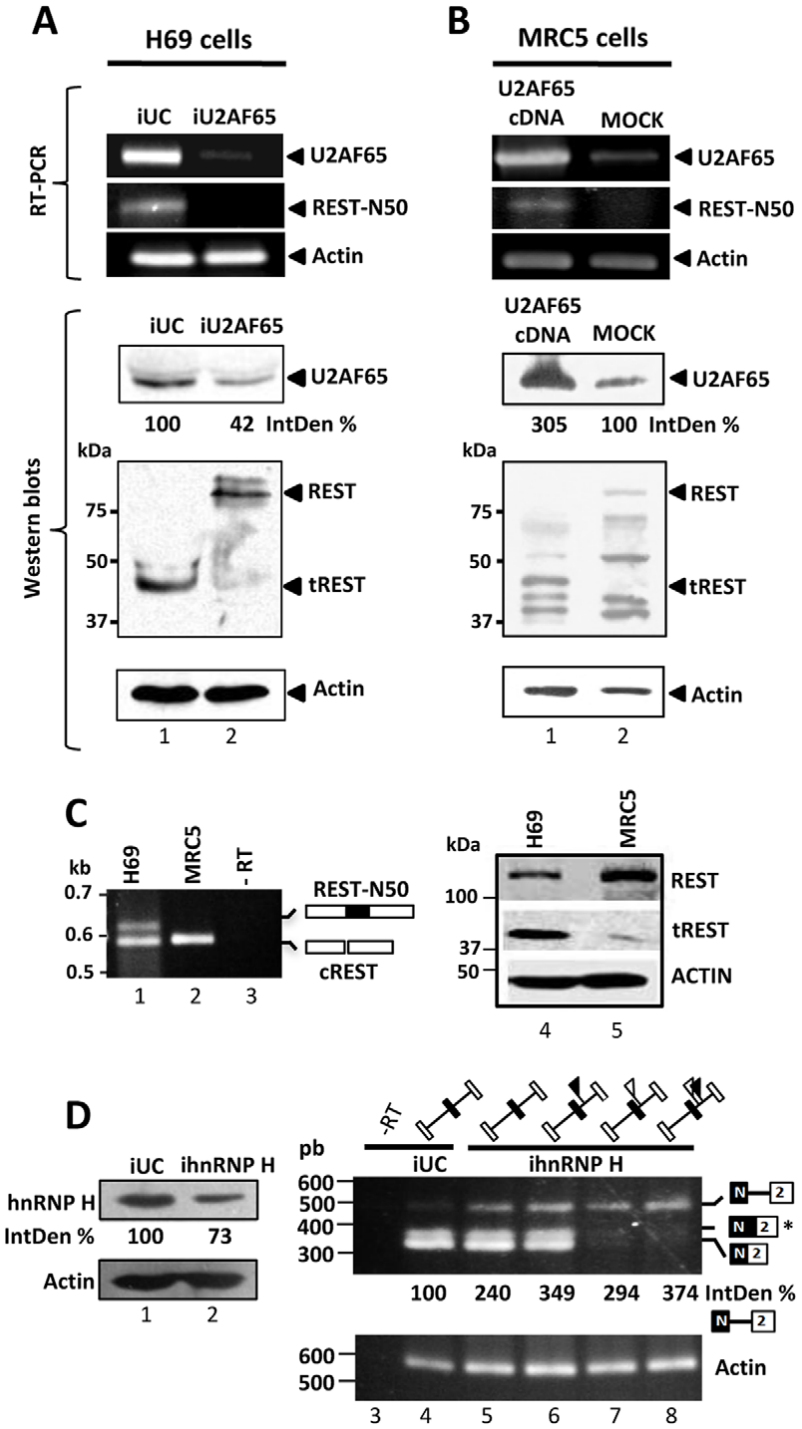
U2AF65 and hnRNP H are required for N50 exon activation in H69 cells. (**A**) **U2AF65 knockdown downregulates REST-N50 mRNA and tREST expression in H69 cells.** H69 cells were treated with universal control (iUC) or U2AF65 siRNAs (lanes 1 and 2, respectively), (**B**) **Overexpression of U2AF65 in MRC5 cells promoted REST-N50 mRNA and tREST expression.** MRC5 cells were transfected with the U2AF65 cDNA (lanes 1) or with empty pcDNA3 vector (lanes 2). 24 h or 36 h post-transfection of siRNAs or cDNAs, respectively, the mRNAs coding for U2AF65, N50-REST, and Actin were monitored by RT-PCR. Protein expression of U2AF65, REST, tREST, and Actin was monitored by western blots as well. (**C**) Steady state expression profiles of REST mRNA variants (lanes 1–3) and protein isoforms (lanes 4–5) in H69 (lanes 1 and 4) and MRC5 (lanes 2 and 5) cells, respectively. No RT (- RT) PCR control reaction appears in lane 3. (**D**) **hnRNP H knockdown leads to accumulation of unspliced N50/β-globin precursor.** H69 cells were treated with iUC (lanes 1, 3 and 4) or hnRNP H siRNAs (lanes 2, 5–8). After 24 h, the cells were transfected with the wild type (lanes 3–5) or mutant N50/β-globin minigenes (lanes 6–8). 24 h post- transfection, N50/β-globin exon 2 splicing was monitored by RT-PCR (with primers SP6 and rtN62). No RT (- RT) PCR control reaction appears in lane 3. hnRNP H knockdown increased the unspliced precursors two-fold. Accumulation of the precursor increased up to 3 times when one or both of the N-PU (black flags) or G2-G3 (white flags) were mutated. Notably, G2-G3 mutants rendered no spliced products, and the N62/2 (*) product was obtained in double transfection experiments only. The PCR products and proteins were quantified densitometrically using the ImageJ software. IntDen % represents the expression change of the indicated protein or mRNA compared to their respective control treatment.

In agreement with their reported functions, we expected that N50 activation and tREST expression could depend on the U2AF65 and hnRNP H splicing promoting activities. To test this possibility we monitored N50 inclusion and the expression of REST isoforms after U2AF65 (Fig. 4A) and hnRNP H (Fig. 4D) knockdown in H69 cells. Compared to the negative control siRNA (Fig. 4A, lane 1), U2AF65 knockdown resulted in N50 skipping, and in reduced tREST expression (lane 2); siRNA treatments did not affect the expression of other splicing (not shown) and housekeeping proteins. Reciprocally, the exogenous U2AF65 expression in MRC5 cells resulted in N50 inclusion, increased tREST expression, and as expected, REST downregulation (Fig. 4B, lane 1). Figure 4C shows the REST mRNA variants and protein isoforms in untreated H69 and MRC5 cells. To test the requirement of hnRNP H, in vivo splicing assays were carried out with the wild type and mutant N exon/β-globin minigenes in hnRNP H-knockdown H69 cells, monitoring the N50/β-globin exon 2 splicing products by RT-PCR. Notably, hnRNP H knockdown resulted in the accumulation of unspliced RNA precursor (Fig. 4D, compare lanes 4 and 5), and the N-PU, G2-G3 and double mutants increased the accumulation of the unspliced precursors even further. Moreover, mutations of the G2-G3 result in only unspliced product, and the splicing of N50 does not occur all. Collectively, these results suggest that U2AF65 and hnRNP H, the N-PU and G2-G3 elements and their respective interactions are essential for N50 exon activation. They also suggest that additional G2-G3-binding factors could be important for N50-5'ss recognition, and that hnRNP H might be a prerequisite for splicing factors recruiting to the N50-5'ss vicinity.

**Figure 5:**
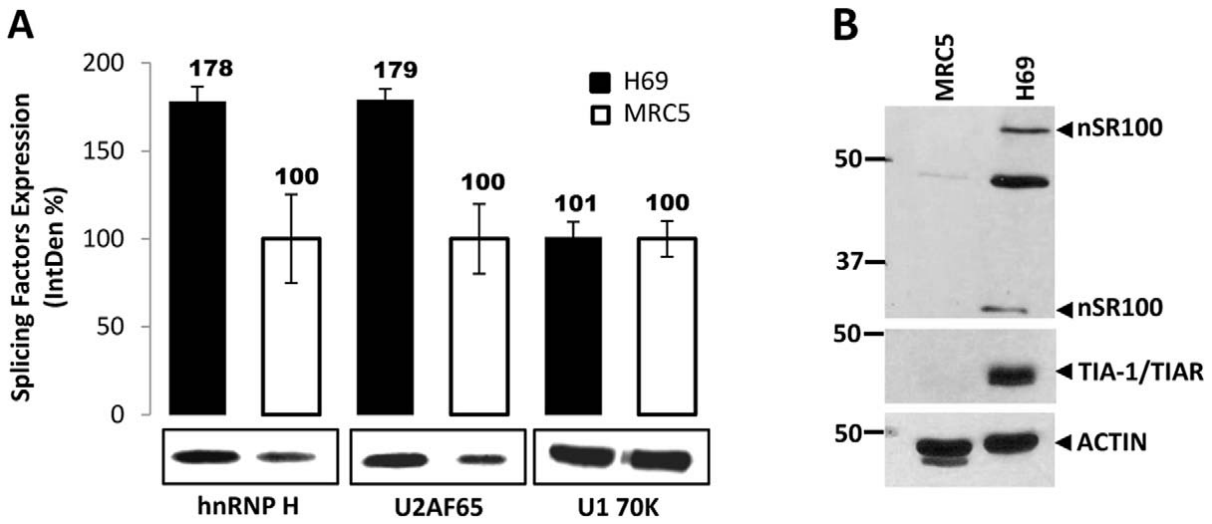
tREST expression correlates with the robust expression of U2AF65, hnRNP H, and other splicing factors in H69 cells. (**A**) The expression of nuclear U2AF65, hnRNP H and snRNP U1 70K in H69 (black bars) and MRC5 (white bars) cells was monitored by western blots. Proteins were quantified densitometrically using the ImageJ software. IntDen % represents the expression change of the indicated protein in H69 cells compared to MRC5 cells. (**B**) Whole cell protein extracts from MRC5 and H69 cell were probed with antibodies against nSR100, TIA-1/TIAR and Actin antibodies. A robust expression of nSR100 and TIAR was detected in H69 cells only.

Confirming our previous findings, and in coordination with the REST-N50 mRNA expression, U2AF65 and hnRNP H expression was more robust in H69 cells (Fig. 5A). As expected, TIA-1/TIAR and nSR100 were highly expressed in H69 cells also (Fig. 5B).

### Mechanistic interplay of U2AF65/N-PU and hnRNP H/G2-G3 in the recognition of the N50-5'ss

**Figure 6:**
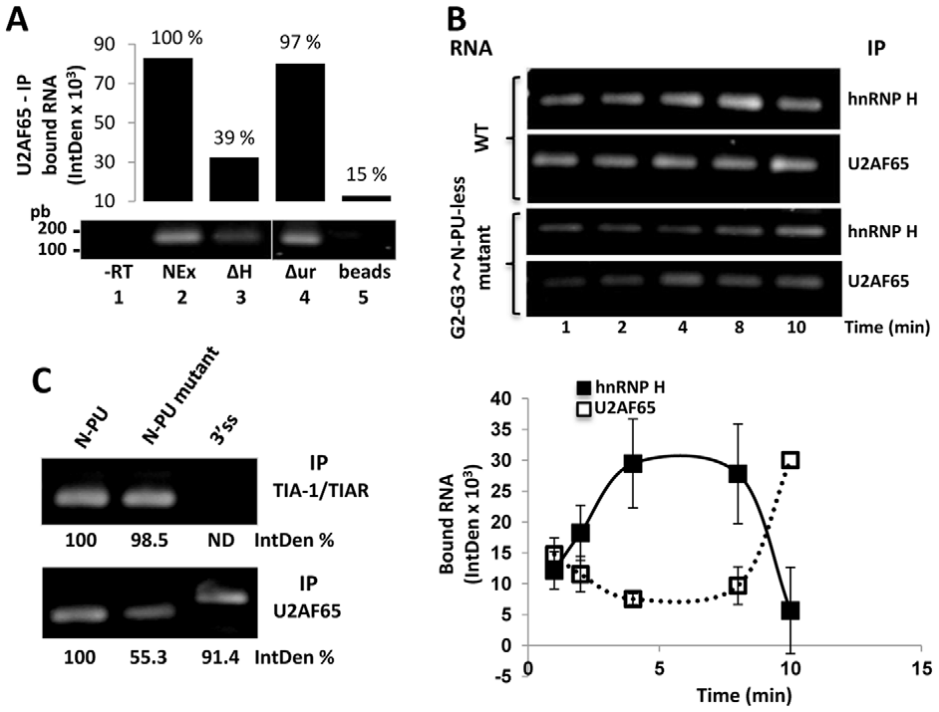
U2AF65/N-PU and hnRNP H/G2-G3 interplay in the N50 exon recognition. (**A**) **hnRNP H-RNA recognition is a prerequisite for U2AF65-RNA binding.** The wild type RNA was incubated with whole (lane 2), hnRNP H-depleted (lane 3), and unrelated antigen-depleted H69 NEx (lane 4) or beads alone (lane 5). Then CLIP assays were carried out using U2AF65 antibodies and the recovered bound RNA was monitored by RT-PCR and quantified densitometrically. No RT (– RT) control PCR appears in lane 1. In the absence of hnRNP H, 60% less RNA was recovered with the U2AF65 antibodies, and no difference was observed in the absence of an unrelated antigen. (**B**) **Kinetics of U2AF65- and hnRNP H-RNA binding.** H69 NEx were incubated with wild type or mutant (G2-G3∼N-PU-less) RNA probes during the indicated times at room temperature. Then, CLIP assays were carried out with U2AF65 or hnRNP H antibodies and the recovered RNAs were amplified by RT-PCR (top panel, electrophoresis of the amplified products) and quantified densitometrically (plot at the bottom). At each time point, IntDen values obtained from the CLIP with the mutant RNA were subtracted from those obtained with wild type RNA in order to discriminate indirect protein-RNA interactions (e.g. mediated by additional factors) and unspecific binding to G-like and PU-like motifs. Significant amounts of hnRNP H-bound RNA are recovered as early as 4 min plummeting at 10 min, coinciding with the onset of U2AF65-bound RNA recovery. (**C**) **TIA-1/TIAR is able to bind to multiple poly-U tracts.** Different probes (wild type G2-G3∼N-PU, G2-G3∼mutant N-PU, and 3'ss control) were used in CLIP experiments as in B. Immunoprecipitations were carried out with antibodies against TIA-1/TIAR and U2AF65, and the recovered RNAs were amplified by RT-PCR and quantified. IntDen % represents the expression change of the indicated protein or mRNA compared to their respective control treatment. ND, not detected.

Several biochemical approaches were used to test the interplay of the nuclear factors U2AF65 and hnRNP H with the N-PU and G2-G3 codes in the recognition of the N50-5'ss. Our previous experiments prompted the idea that RNA-bound hnRNP H might be a prerequisite for the recruitment of other nuclear factors to the N50-5'ss (Figs. 3B and 4D). To gain insights on this possibility, CLIP assays were carried out with the G2-G3∼N-PU probe and whole or hnRNP H-depleted NEx (Fig. S3) from H69 cells, that were immunoprecipitated with U2AF65 antibodies. The recovered wild type RNA was amplified by RT-PCR and quantified. The absence of hnRNP H affected U2AF65-bound RNA recovery by 60% (Fig. 6A, lane 3), whereas no significant binding reduction was observed when the extracts were depleted with an unrelated antibody (lane 4). These, and previous results (Figs. 2 and 4) support the notion that hnRNP H bound to the G2-G3 elements might be a prerequisite for the recognition of the NP-U by U2AF65.

Therefore, similar experiments were carried out to explore the kinetics of hnRNP H and U2AF65 binding to the N exon wild type RNA. Given that additional G-rich and uridine-rich elements are present in the RNA and that the CLIP assays were carried out with whole NEx, to discriminate stable and direct (i.e. not mediated by other nuclear factors) protein/RNA interactions, the signals of mutant RNAs lacking the G2-G3 and N-PU elements were subtracted from those of the wild type RNA. The RNA immunoprecipitated with hnRNP H peaked between 4–8 min, and plummeted at 10 min (Fig. 6B). However, as expected from the different protein isoforms and differential RNA structure recognition [20], small amounts of RNA were immunoprecipitated with U2AF65 throughout, reaching a maximum at 10 min (Fig. 6B). These data might reflect that hnRNP H recognizes the G2-G3 elements early during complex assembly, and that either by protein isoform substitution or RNA structure stabilization, U2AF65 binding to the N-PU is delayed. Furthermore, these data also suggest that U2AF65 RNA-binding displaces hnRNP H binding to the juxtaposed G2-G3 elements.

Since TIA-1 and TIA1-like proteins are able to bind to U-rich motifs downstream of 5'ss facilitating its recognition [9] and that these proteins are abundantly expressed in H69 cells, CLIP assays were carried out to investigate whether these proteins were able to bind to the N-PU. Similar amounts of wild type- and mutant-N-PU RNAs were recovered with the TIA-1/TIAR antibodies (Fig. 6C), suggesting that these proteins bind to one or more upstream U-rich motifs, different to the N-PU. No RNA was recovered with a 3'ss control probe.

**Figure 7:**
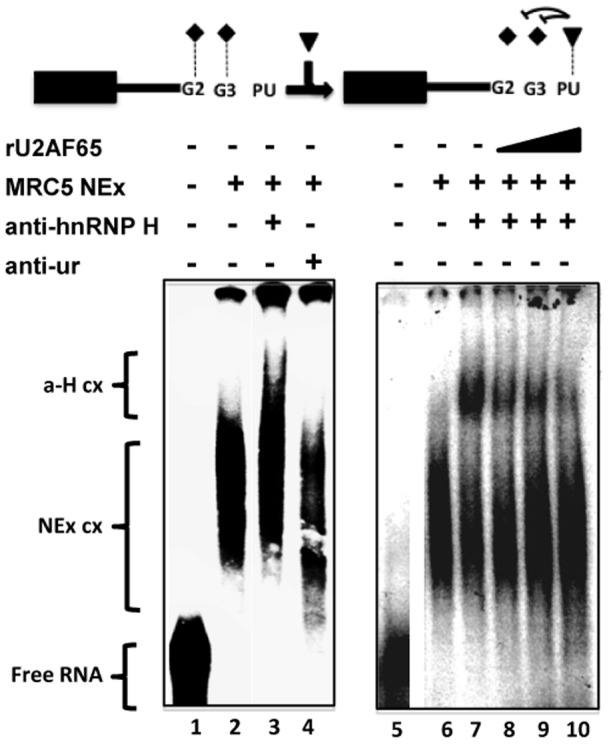
U2AF65/N-PU recognition displaces hnRNP H binding to the G2-G3 motifs in a dose dependent manner. Supershift experiments were carried out with the G2-G3∼N-PU probe (free probes are in lanes 1 and 5), NEx from MRC5 cells (lanes 2–4, and 6–10), anti-hnRNP H antibodies (lanes 3, 7–10), antibodies against an unrelated antigen (lane 4), and increasing amounts of rU2AF65 (2, 4, and 6 µg; lanes 8–10, respectively). The complexes supershifted by the hnRNP H antibody (a-H cx) migrated back to the original position of the NEx complexes (NEx cx) as the amount of rU2AF65 increased. Probe and factors depicted as in Fig. 1C.

In view of the above results, and that binding of U2AF65 to pyrimidine-rich sequences in front of a 5'ss can activate the ss by means of U1 snRNP recruiting [11], it is possible that to exert its 5'ss activating function, U2AF65 might displace hnRNP H from G2-G3 by a steric competitor effect. To investigate this, the complexes formed with the wild type probe and MRC5 NEx (Fig. 7, lanes 2 and 6), were super-shifted with anti-hnRNP H antibodies (lanes 3 and 7), but not with antibodies against unrelated antigens (lane 4). However, super-shifting of the complexes was challenged with increasing amounts of rU2AF65 in a dose dependent manner (Fig. 7, lanes 8–10, respectively), suggesting that U2AF65 sterically hinders hnRNP H binding to the intronic G2-G3 elements.

**Figure 8:**
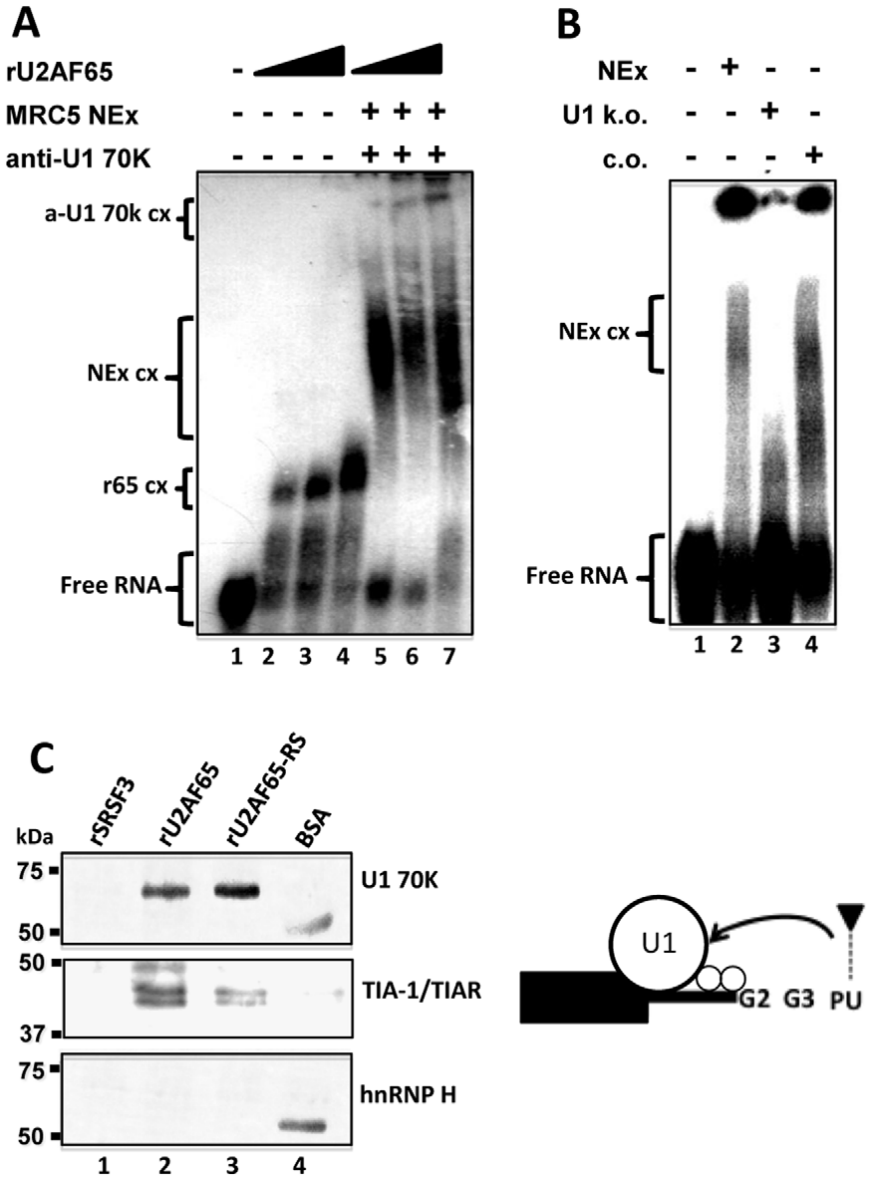
The RS domain of U2AF65 promotes U1 snRNP recruitment to the N50-5'ss. (**A**) The G2-G3∼N-PU probe (free probe in lane 1) was used in EMSA experiments with increasing amounts of rU2AF65 (1–3 µg) forming the complexes r65 cx (lanes 2–4, respectively). The same probe was incubated with MRC5 NEx forming the complexes NEx cx (lanes 5–7). NEx cx were supershifted with anti-U1 70K antibodies (a-U1 70k cx) in the presence of 1–3 µg of rU2AF65 (lanes 5–7, respectively). The amount of a-U1 70k cx increased with the amount of rU2AF65. (**B**) The same probe (free probe, lane 1) was used in EMSA experiments with NEx from H69 cells (lane 2), or with extracts incubated with oligonucleotides targeted to the 5' end of the U1 snRNA and treated with RNase H (U1 k.o.; lane 3), or with RNase H-treated extracts incubated with scrambled oligonucleotides (c.o.; lane 4). Probe and factors depicted as in Fig. 1C. (**C**) Nickel or protein-A Sepharose (S) beads covered with rSRSF3-(S), rU2AF65, and the SR domain of U2AF65 (rU2AF65-RS) were used in pull-down experiments. U1 70K, TIA-1/TIAR, and hnRNP H (top, middle, and bottom panels, respectively) were monitored by western blots. Both snRNP U1 70K and TIA-1/TIAR were able to interact with U2AF65 and its RS domain (lanes 2, and 3) but not with a different splicing protein, rSRSF3 (lane 1). BSA covered beads were used for blank controls (lane 4); in these lanes protein-A copurified with BSA. No hnRNP H was pulled-down with rU2AF65. The cartoon depicts the possible U1 snRNP and TIA-1/TIAR interactions with U2AF65 in the recognition of a 5'ss.

The activating role of U2AF65 was explored further. Mimicking H69 and MRC5 cellular contexts, U2AF65-depleted H69 NEx was not as efficient as whole NEx to recruit EMSA complexes with the G2-G3∼N-PU probe, and the complexes formed with the same probe and MRC5 NEx were supershifted with rU2AF65 (Fig. S4). This suggested that U2AF65 could participate in recruiting other splicing factors to the N50-5'ss. Recombinant U2AF65 was able to form complexes with the G2-G3∼N-PU probe in a dose dependent manner (Fig. 8A, lanes 1–4), and larger complexes were formed with MRC5 NEx in the presence of U1 70K antibodies (lane 5). The signal of the supershifted complexes increased with the amount of rU2AF65 (lanes 6–7), suggesting that U2AF65 might facilitate U1 snRNP recruitment to the N50-5'ss as previously described [11]. In addition, efficient complex formation was observed with the probe and NEx from H69 cells, but not with extracts in which the 5' end of U1 snRNA was degraded by RNase H-mediated digestion with a complementary oligonucleotide (Fig. 8B, lanes 1–3, respectively); the use of a scrambled oligonucleotide as a control for U1 snRNA degradation did not affect complex formation (lane 4). Furthermore, pull-down assays were carried out with NEx from H69 cells and Sepharose beads covered with rSRSF3, rU2AF65, the rU2AF65-RS domain or BSA, and the precipitated proteins were detected by western blot. U2AF65 and the rU2AF65-RS domain (Fig. 8C, lanes 2 and 3, respectively) were able to pull-down U1 snRNP 70K and TIA-1/TIAR. No U1 snRNP 70K was pulled-down with the RS domain of rSRSF3 or BSA-covered beads (Fig. 8C, lanes 1 and 4, respectively). Conversely, rU2AF65 did not recruit hnRNP H (bottom panel). Altogether, the results suggest that U2AF65 binding to the N-PU hinders hnRNP H G2-G3 recognition and promotes, together with TIA-1/TIAR, U1 snRNP 70K recruitment to the 5'ss.

## Discussion

### The role of the REST protein in the phenotype of H69 cells

REST expression profiles in H69 cells directly affect the determination of the transformed phenotype. In fact, transfection of the N50-REST cDNA into MRC5 cells modifies the expression profiles of the REST target genes RRAD (Ras-related associated with diabetes) and TMS-1 (target of methylation induced silencing-1) (Fig. S5). In this respect, it has been suggested that truncated REST isoforms function by antagonizing cREST; however their roles seem to be much more diverse. The fact that REST4 has not direct effect on the activation or repression of synapsin I in mouse NS20Y cells [21], suggests a different role for this splicing variant instead of antagonizing REST function, or directly activating REST target genes [22], [23]. Alternatively, an increasing number of REST-related small RNAs and the many profiles of REST interactions could explain new roles for REST neuronal isoforms [24], [25]. Whereas constitutively expressed REST suppresses a vast number of the neuronal genes in non-neuronal tissues [26], [27], a decrease of REST is essential for establishing and maintenance of neuronal fates. In fact, REST is post-translationally downregulated in early stages of neurogenesis allowing the expression of neuronal genes [28]. Concomitant with the REST degradation, the persistence of REST transcripts after protein degradation in neurogenesis and adult neuronal tissues correlates with truncated REST (mRNA) variants, suggesting that the solely REST degradation is not sufficient for the acquisition of neuron fates [6], [28]. Recently, however, Raj et al. [19] showed that neurogenesis is controlled by a cross-regulation between nSR100 and REST.

As previously demonstrated by Coulson and coworkers [4], here we observed mRNA and REST protein as well as the splicing variant REST-N50 in H69 cells, cell that express neuronal traits (Fig. 4). However, works from Neumann and coworkers failed to detect either the mRNA or REST protein suggesting a transcriptional downregulation of REST and the consequent shortage of REST protein [29]. Our experiments demonstrated the presence of REST and tREST and evidenced rapid degradation of REST after translation inhibition with emetine as reported to occur during neurogenesis process [28]. Moreover, we found REST stabilization in MG132 treated H69 cells suggesting that REST is degraded via proteasome (Fig. S6). Interestingly, tREST expression remained in translation inhibition experiments even after long time; although other possibilities may explain tREST endurance, it is possible that this isoform does not undergo proteasome degradation due to its lack of many potential ubiquitination sites. Also, in agreement with previous findings [19], REST downregulation may also obey to the robust expression of nSR100 in H69 cells.

### N50 splicing mechanism in H69 cells

Previous works have identified multiple 5'ss surrounding the REST N exon, suggesting tissue-specific activation profiles [5], [6]. Our findings are in agreement with the N50 exon sequence features which reflect the preferred selection of the N50-5'ss over the remaining alternative ss. The N50-5ss is strong and is surrounded by U- and G-rich tracts (e.g. the N-PU and G2-G3 codes), located downstream the N50-5'ss, which resemble those found in many neuronal exons [9], [10], [14]. We have shown that N50 exon inclusion depends on the combinatorial function of a complex array of splicing regulatory signals and robust expression of U2AF65, hnRNP H and other splicing factors (Figs. 2, 4, and 5). Interestingly, mutations of the G2-G3 codes had a stronger negative effect on N50 exon inclusion than N-PU mutants (Figs. 2 and 4D), suggesting that hnRNP H binding to the G2-G3 has a more relevant role in N50 exon activation. These findings also suggested that hnRNP H and other factors binding to and nearby the G2-G3 occurred first, and that could facilitate U2AF65 recruitment to the N-PU. Although other factors are found in the RNA complexes, the biochemical data showed direct U2AF65/N-PU and hnRNP H/G2-G3 RNA-protein binding (Figs. 3, and 6). We also observed more pronounced hnRNP H supershift signals in G1-containing RNAs (Fig. S7). Since the G1 is juxtaposed to the N4-5′ss, it is probable that hnRNP H and additional factors recruited in the G1 element block the N4-5'ss.

The binding and in vivo splicing assays (Figs. 2, 4D, and 6A) indicated that hnRNP H and the G2-G3 codes are essential and a prerequisite for U2AF65 recruitment to the N-PU and N50 exon inclusion. In agreement with this, the time course experiments (Fig. 6B) strongly indicated that hnRNP H initially forms stable RNA-protein complexes, followed by U2AF65 recruitment to the N-PU and hnRNP H dissociation. It has been argued that U2AF65 initially binds to the RNA and that such complex undergoes additional conformational rearrangements to maximize binding interaction, thus entailing multiple adjustment stages [20]. Therefore U2AF65-RNA associations at short times (4 – 8 min) may be transient and attributed to both protein and RNA conformational dynamics, or to a possible interplay between many other factors. It is possible that the hnRNP H dissociation after U2AF65 binding may be caused by steric hindrance or by the RNA refolding, due to juxtaposition of the G2-G3 and the N-PU elements.

U1 snRNP is the first sub complex thought to bind the 5'ss during the spliceosome assembly. It is accepted that many auxiliary-splicing factors can promote weak 5'ss recognition by binding to enhancer sequences [30], [31]. Despite of U2AF65 interactome is thought to be large, little has been reported concerning U2AF65-U1 snRNP interactions [11], [32]. Forch and colleagues showed that the in vitro U2AF65 binding to a PU tract near to the 5'ss of the mls-2 RNA from *Drosophila* can promote U1 snRNP recruitment [11]. In agreement with this, we found that the presence of the U1 snRNP in the supershifted complexes was proportional to the amount of rU2AF65 suggesting U2AF65 requirement (Fig. 8) and that depletion of U2AF65 diminishes complex formation (Fig. S4). In addition, in U1 snRNA-depleted H69 NEx no evident complex formation was observed indicating the requirement of the U1 snRNA for N50-5'ss and U1 snRNP association (Fig. 8B). U2AF65 depletion in these experiments mimicked U1 snRNA degradation, reinforcing U1 snRNP recruitment via U2AF65 (Fig. S8). Furthermore, pull down assays showed that the RS domain of U2AF65 is enough to recruit the U1 snRNP. Given the robust expression of TIA-1/TIAR in H69 cells, and that TIA-1/TIAR binds poly-U tracts nearby the G2-G3 elements, and because it precipitates in U2AF65 pull-down experiments, it is possible that both TIA-1/TIAR and U2AF65 actively participate in U1 snRNP recruitment to the N50-5'ss.

Collectively, our results suggest that the initial hnRNP H binding to the G2-G3 codes is a prerequisite for U2AF65 recruiting to the N-PU tract. Then U2AF65 along with other factors might help U1 snRNP recruitment to the N50-5'ss for N exon activation.

We found here that N exon is a very versatile sequence with a complex array of splicing signals, and its activation in H69 cells depends on the relative amounts of hnRNP H and U2AF65. Although further studies are needed to unveil the activation of the N4- and N62-5'ss, we gained some insights in a novel-activating role of hnRNP H, being a prerequisite for U2AF65 recruiting to nearby sequences.

The U2AF65 hnRNP H-displacing function evidenced here is new, and might have been acquired early in evolution. This particular function resembles that of SRSF1 and SRSF2 that bind to juxtaposed GGGG codes downstream of the chicken tropomyosin exon 6B displacing hnRNP A1 from these sites and promoting exon inclusion [33]. Also, such function resembles that of hnRNP A1 when displacing U2AF65 from non-AG-containing/uridine-rich 3'ss [34]. This is the first report on the recruitment of U2AF65 downstream of the N50-5'ss for its activation in a human pre-mRNA.

Finally, we show here precedents for the repressing and recruiting functions of hnRNP H (in a consecutive fashion) and U2AF65 (in a simultaneous manner) along the splicing mechanisms associated with cancer cells.

## Materials and Methods

### Cell cultures and nuclear extracts

The human H69 (SCLC), A549 (lung adenocarcinoma epithelial; used in supplementary experiments), and MRC-5 (normal diploid secondary lung fibroblasts) cells were from ATCC (Manassas, VA, USA). Cells were cultured with RPMI and DMEM media, respectively, both supplemented with 10% fetal bovine serum. Nuclear extracts were made according to the Dignam protocol [35].

### PCR and Computational tools

H69 cells genomic DNA containing 593 nt around the N exon was amplified by PCR, using primers Seq1s and Seq1as (primer sequences and PCR conditions are listed in Fig. S1). The amplified DNA was sequenced and 163 nt of the N exon and intron sequences were analyzed for ss strength using the on-line Alternative Splice Site Predictor (University of Sevilla). The tool uses pre-processing models for position-specific score matrixes and a backpropagation network for the positive hits. Therefore, low scores represent false ss, and values above the cutoff result in reduced detection of alternative isoforms and cryptic ss. The Splicing Rainbow was used to search for putative binding sites for splicing factors.

### siRNAs, constructs, transfections, western blotting and densitometries

The pre-characterized U2AF65 (HSS117616), hnRNP H (HSS104905), and negative universal control (46-2001) siRNAs were purchased from Invitrogen. To construct the wt and mutant REST β-globin-N exon minigenes we first amplified the first two exons and intron of the β-globin gene with primers gloF and gloR. The fragment was cloned in the pCR2.1 plasmid. Next, the N exon sequencing fragment was used as template to amplify the wt, PU mut, G2-G3 mut and double mutant N exons using the forward primer 2REN/IVs and the reverse primers PU-rev, PU mut, G2G3m-as, and d-mut, respectively. Each fragment was inserted into the Bbs I site of the β-globin intron of the pCR2.1 clone, and each intermediary was subcloned into the pcDNA3 plasmid digested with Kpn I and Xho I. Clones were verified with Nsi I digests and sequencing. Plasmids were purified with Plasmid Midi Kit (QIAGEN), and transfected into human cells. The primers U2AF65F and U2AF65R were used to amplify de cDNA of U2AF65 by RT-PCR. The fragment was cloned into the Bam HI and Xho I sites of the pPROEX HTb plasmid. To maintain the RS domain of U2AF65, the Hind III-Sma I fragment was deleted by religation. Likewise, to maintain the SR domain of SRSF3, the Sma I-Xho I fragment was deleted from a cDNA SRSF3 clone in pGEX-2T. For overexpression experiments, the cDNA of U2AF65 was cloned in the plasmid pcDNA3.1. For transfection experiments, plasmids (2 μg) or siRNAs (20 nM) were transfected with Lipofectamine 2000 in six-well culture plates following the manufacturer's instructions (Invitrogen), and mRNA and protein expression were analyzed. Total RNA was prepared with RNasy Kit following the manufacturer's instructions (Qiagen). For reverse transcription, Superscript II (Invitrogen) was used. For H69 and MRC-5 whole-cell extracts, cells were disrupted in lysis buffer (10 mM NaCl_2_, 10 mM Tris.HCl, pH 7.5, 1.5 mM MgCl_2_ and 1% NP-40), and 10 μg were resolved in 10% polyacrylamide gel. Antibodies against REST, hnRNP H, hnRNP F, pan-SRSF, U2AF65, U1 70K (Santa Cruz Biotechnology, Inc.), and Actin (a gift from J.M. Hernández) were used for western blotting, CLIP, supershift, and pull-down assays. Digital image densitometries of PCR products and protein bands were carried out, from at least three experiments each, with the ImageJ software and expressed as Integrated Density (IntDen) or its percent.

### RNA labeling

DNA templates were amplified by PCR. For UV crosslinking and supershift assays, RNAs carrying the exon N were transcribed in vitro using the Riboprobe® System-T7 (PROMEGA) as instructed by the manufacturer, RNAs were purified from a 5% denaturing acrylamyde gel using the RNA elution buffer (0.3 M sodium acetate, 0.2% SDS). RNA was extracted with phenol:chloroform:isoamyl alcohol and ethanol precipitated.

### UV crosslinking and crosslinking-immunoprecipitation (CLIP)

One µg of NEx was incubated with 200 fmole of ^32^P-labeled RNA for 30 min at 4°C in splicing buffer (5mM HEPES pH 7.9, 0.6% polyvinyl alcohol, 20 mM creatine phosphate, 10% glycerol and 0.4 mM ATP). After incubation RNA-bound NEx were UV irradiated for 20 min at a wavelength of 254 nm on ice, then incubated with 0.5 µg of RNAse A for 30 min at 37°C, and immunoprecipitated as described [36], or treated for electrophoresis. In some cases 10 nM Psoralen was added in the UV crosslinking reaction. For RNA binding assessments, CLIPs were carried out with cold RNA and precipitates were washed 3 times with washing buffer. Pellets were treated with Proteinase K-SDS and the bound RNA probe was extracted with phenol-chloroform and PCR-amplified with primers N4T7-fwd and PU-rev.

### EMSA and protein purification from RNA-protein complexes

Splicing reactions were assembled in 10 μl of binding buffer (50 mM Hepes-KOH, pH 7.6, 40 mM MgCl_2_, 20 mM spermidine, 0.1 mM DTT, 0.5 mM EDTA, 1.5 mM ATP and 20% Glycerol) containing 10 μg of nuclear extract and 0.4 μg/μL of tRNA, and were incubated for 15 minutes at room temperature (r/t). 2000 cpm of ^32^P-labelled substrate RNA was added and incubated for 15 minutes at r/t. Samples were loaded on a 4.0% native polyacrylamide gel, electrophoresed, and visualized by autoradiography. Proteins were purified from sliced RNA-protein complexes as previously described [37].

### rU2AF65 expression and purification

Plasmids pProEXHTb-U2AF65 (or the RS domain of U2AF65) were transformed into *E. coli* BL21, incubated at 37°C (OD = 0.6–0.8) and induced with IPTG 0.2 mM during 4 hours. Proteins were isolated from bacterial pellets in denaturing conditions, as indicated by the manufacturer (the QIAexpressionist^TM^), and renatured by sequential dialysis in buffers with decreasing amounts of urea, and later incubated on ice overnight. Glycerol (20%) and protease inhibitors were added. Proteins were stored at −70°C until use.

### Pull-down assay

Ten µg of 6xHis-tagged protein (U2AF65-RS domain or full-length) were bound to Ni-NTA beads in binding buffer (10 mM Tris, 100 mM N_9_H_2_PO_4_.H_2_O, pH = 8.0) for 1 h at r/t. Beads were collected by centrifugation, washed three times with PBS and incubated 30 min in 300 µl of 10% Bovine Serum Albumin (BSA) at 4°C. The beads were then incubated overnight at 4°C with 200 µg of H69 NEx in 400 µl of PBS and unbound proteins were washed three times with PBS by centrifugation. Bound proteins were identified by western blot.

## Supporting Information

Figure S1 **Oligonucleotides used in this work.** Primers used to amplify the REST, RRAD, TMS-1 and Actin cDNAs. The GA3PDH cDNA was amplified with primers reported by Choi-Lundberg and Bohn. 1995. Brain Res. Dev. Brain Res. 85 (1): 80-8. The primers used to amplify the DNA templates for run-off transcription; sequencing and plasmid constructions are also shown. The PCR amplification conditions were preceded and followed by a denaturing cycle at 94°C for 5 min, an extension cycle at 72°C for 7 min, respectively.
(PDF)

Figure S2 **The N-PU is a bona fide poly-Uridine tract recognized by U2AF65 from H69 cells.** The wild type N-PU (lanes 1–4) or mutant N-Pn (lanes 5–8) RNAs (shown at the top) were used in EMSA experiments with 2 µg (lanes 2 and 6), 4 µg (lanes 3 and 7) or 6 µg (lanes 4 and 8) of rU2AF65; free probes were run in lanes 1 and 5, respectively. The two remaining uridines in the mutant RNA (UUUUUU to GAUAUC) account for the residual (roughly 20%) rU2AF65 complexes (r65 cx), suggesting that the N-PU is necessary for U2AF65 recruiting to the N exon alternative 5'ss. An empty arrowhead indicates a structured form of the mutant probe.
(PDF)

Figure S3 **hnRNP H immunodepletion.** NEx from H69 cells were depleted using antibodies against an unrelated antigen (Δur) or against hnRNP H (ΔH). hnRNP H antibodies were used to probe western blots after immunodepletion. The hnRNP H (P H) and unrelated proteins (P ur) bound to the pellets after immunodepletions were analyzed also. To control the hnRNP H depletion, the same membrane was stripped and incubated with antibodies against U2AF65 (lower panel). An asterisk indicates residual hnRNP H after stripping.
(PDF)

Figure S4 **U2AF65 is necessary for complex recruiting at the N50-5**'**ss.** The wild type probe (shown at the top; lanes 1 and 5) was used in EMSA with NEx from H69 cells (lane 2), or with H69 NEx depleted of U2AF65 (ΔU2AF65; lane 3) or depleted of an unrelated antigen (Δunrel; lane 4). Complex formation was reduced with the ΔU2AF65 NEx. Reciprocally, the probe was incubated with NEx from MRC5 cells alone (lane 6) or complemented with rU2AF65 (lane 7). rU2AF65 was required for shifting the mobility of the complex and the remaining probe, in a different manner than rU2AF65 alone (lane 8).
(PDF)

Figure S5 **Transfection of the cDNA for tREST into MRC5 cells modifies RRAD and TMS-1 basal expression.** (**A**) Putative binding sites for REST were localized close to the TATA box in the promoters of the human RRAD and TMS-1 genes. (**B**) RT-PCR showing the altered expression of RRAD and TMS-1 in cells transfected with the tREST cDNA (lane 2), compared to the MOCK transfections (lane 3), and no RT controls (lane 1). The GA3PDH amplicon was used for loading control.
(PDF)

Figure S6 **cREST is readily degraded in H69 cells.** (**A**) Western blots from emetine-treated (+; 100 μg/mL) or untreated (-) H69 (upper panel) and MRC5 cells (lower panel). Incubation times were 1, 5 and 10 h. Canonical (cREST) and truncated (tREST) isoforms are indicated. (**B**) Nuclear extracts from H69 cells treated (+) or not (-; 0.2% DMSO only) with the proteasome inhibitor MG132 at 10 μM final concentration during 4 h were probed with anti-REST antibody. REST (arrowhead in lane 1) and ubiquitinated-REST (brackets in lane 2) are shown.
(PDF)

Figure S7 **hnRNP H from H69 cells is able to bind to all G codes.** (**A**) Twenty fmole of a ^32^P-labeled RNA probe (shown on top) containing part of the N4-5ss only, the 3'ss, the Py and part of the upstream intron were incubated with 5 µg of each NEx from MRC5, A549 (other SCLC cell line) or H69 cells. The EMSA complexes were resolved in a 4% native polyacrylamide gel. Proteins from 20 complexes were purified and visualized in silver stained 10% SDS-polyacrylamide gels. Black arrows show the proteins that were differentially purified from NEx. (**B**) A sample of the purified complexes was electrophoresed and probed with anti-hnRNP H antibodies by western blot. A strong signal for hnRNP H was detected in MRC5 (lane 1) and H69 (lane 3) cells indicating that hnRNP H is recruited to the G1. A less intense hnRNP H signal was observed in A549 NEx. (**C**) Two different RNA probes (drawings at the bottom), with or without the G1 element, were labeled (lanes 1 and 5, respectively) and used in EMSA with H69 NEx. Both probes rendered complexes with the NEx (lanes 2 and 6) but that with the G1 element was supershifted more efficiently with the anti-hnRNP H antibodies (lane 3) as compared with the modest supershift obtained with the probe lacking the G1 element (lanes 7 and 8; antibody added before or after the probe). Antibodies against hnRNP A did not supershifted the complexes formed with either probe.
(PDF)

Figure S8 **Depletion of U1 snRNA or U2AF65 of H69 NEx inhibits complex recruiting around the N exon.** The wild type probe (lane 1) was used in EMSA with NEx from H69 cells (lane 4), or with H69 NEx depleted of U2AF65 (ΔU2AF65; lane 5) or with NEx in which the 5' end of U1 snRNA was degraded by RNase H-mediated digestion with a complementary oligonucleotide (NEx U1 k.o.; lane 3). Complex formation was not diminished when the U1 snRNA was degraded with a scrambled oligonucleotide as a control (NEx c.o.; lane 2) and was partially recovered when the ΔU2AF65 extracts were supplemented with recombinant U2AF65 (Δ65 + rU2AF65; lane 6).
(PDF)
