# Supplementary material for: Binding of hnRNP H and U2AF65 to Respective G-codes and a Poly-Uridine Tract Collaborate in the N50-5'ss Selection of the REST N Exon in H69 Cells
Source: PLoS One. 2012 Jul 5;7(7):e40315. doi: 10.1371/journal.pone.0040315 (PMC3390395; doi:10.1371/journal.pone.0040315)
Supplement: Figure S1 — Oligonucleotides used in this work. Primers used to amplify the REST, RRAD, TMS-1 and Actin cDNAs. The GA3PDH cDNA was amplified with primers reported by Choi-Lundberg and Bohn. 1995. Brain Res. Dev. Brain Res. 85 (1): 80-8. The primers used to amplify the DNA templates for run-off transcription; sequencing and plasmid constructions are also shown. The PCR amplification conditions were preceded and followed by a denaturing cycle at 94°C for 5 min, an extension cycle at 72°C for 7 min, respectively. (PDF) [file pone.0040315.s001.pdf]

GENE AMPLIFIED	OLIGONUCLEOTIDE SEQUENCE (5' ➔3')	NAME	PCR PARAMETERS	PRODUCT LENGTH
FOR PCR				
REST	GCAGGAGAACGCCCATATAAATGTG<br>CTGGTAGTCACCTGGATCACTGAGAC	V-fwd<br>VI-rev	94°C/30 s, 64°C/30 s, 72°C/60	578 + 628
REST-N50	GCAGGAGAACGCCCATATAAATGTG<br>CCTGAATACATACCCATCTAGATC	V-fwd<br>N-rev	94°C/30 s, 58°C/30 s, 72°C/30 s	143
TMS1	TGGGCCTGCAGGAGATG<br>ATTTGGTGGGATTGCCAG	TMS1-fwd<br>TMS1-rev	94°C/30 s, 54°C/30 s, 72°C/30 s	410
RRAD	TTTACAAGGTGCTGCTGCTGGG<br>TGCCGCTGATGTCTCAATGAAC	RRAD-fwd<br>RRAD-rev	94°C/30 s, 64°C/45 s, 72°C/60 s	433
ACTIN	GTGGGGCGCCCCAGGCACCA<br>CTCCTTAATGTCACGCACGATTTC	ACT-fwd<br>ACT-rev	95°C/45 s, 55°C/45 s, 72°C/30 s	523
β-globin minigene	ATTTAGGTGACACTATAG<br>TAATACGACTCACTATAG	SP6<br>T7	95°C/45 s, 55°C/45 s, 72°C/30 s	556
N/2	ATTTAGGTGACACTATAG<br>CCATTTGGTAATATTTACTAGAGTGT	SP6<br>rtN62	95°C/45 s, 55°C/45 s, 72°C/30 s	356
FOR TRANSCRIPTION				
REST DNA TEMPLATE	GGAGAAGAATTGTCCAGTCAGCTG<br>CTGGTAGTCACCTGGATCACTGAGAC	Int 6-fwd<br>VI-rev	94°C/30 s, 64°C/30 s, 72°C/60	2953
flWT PROBE	TAATACGACTCACTATAGGGACCAGTGGGGTATGGATACCATTTGG<br>TACATAAAAAACCAGAACCCCCACCCCC	N4T7-fwd<br>PU-rev	94°C/30 s, 64°C/30 s, 72°C/60	125
WT PROBE	TAATACGACTCACTATAGCTACAGTGTGATCTAGATGGGTATG<br>TACATAAAAAACCAGAACCCCCACCCCC	N50T7-fwd<br>PU-rev	94°C/30 s, 64°C/30 s, 72°C/60	90
PU MUT used in SF5	TAATACGACTCACTATAGCTACAGTGTGATCTAGATGGGTATG<br>TACATGATATCCCAGAACCCCCACCCCC	N50T7-fwd<br>PU mut	94°C/30 s, 64°C/30 s, 72°C/60	90
PROBE used in SF3	TAATACGACTCACTATAGGGACCAGTGGGGTATGGATACCATTTGG<br>CCTGAATACATACCCATCTAGATC	N4T7-fwd<br>N-rev	94°C/30 s, 64°C/30 s, 72°C/60	70
FOR SEQUENCING AND CONSTRUCTS				
N EXON SEQ/ TEMPLATE	TGCTCAAAGTCTCAGAGTGAG<br>TACATAAAAAACCAGAACCCCCACCCCC	Seq1s<br>PU-rev	94°C/30 s, 64°C/45 s, 72°C/60 s	593
WT N EXON	GGAGAAGAATTGTCCAGTCAGCTG<br>TACATAAAAAACCAGAACCCCCACCCCC	2REN/IVs<br>PU-rev	94°C/30 s, 64°C/45 s, 72°C/60 s	358
N EXON PU mut	GGAGAAGAATTGTCCAGTCAGCTG<br>TACATGATATCCCAGAACCCCCACCCCC	2REN/IVs<br>PU mut	94°C/30 s, 64°C/45 s, 72°C/60 s	358
N EXON G2G3 mut	GGAGAAGAATTGTCCAGTCAGCTG<br>TACATAAAAAACCAGAACCGTGACCGTG	2REN/Ivs<br>G2G3m-as	94°C/30 s, 64°C/45 s, 72°C/60 s	358
N EXON d mut	GGAGAAGAATTGTCCAGTCAGCTG<br>TACATGATATCCCAGAACCGTGACCGTG	2REN/IVs<br>d-mut	94°C/30 s, 64°C/45 s, 72°C/60 s	358
GLOBIN	CCTCAAACAGACACCATGGTGC<br>CCCTGAAGTTCTCAGGATCCACG	gloF<br>glotR	94°C/30 s, 64°C/30 s, 72°C/60 s	529
U2AF65	CTCGAGCTACCAGAAGTCCCGGCG<br>GGATCCAGTCGGACTTCGACGAGTTC	U2AF65F<br>U2AF65R	95°C/30 s, 65°C/60 s, 72°C/150 s	1488
