# Supplementary figures and images for: Binding of hnRNP H and U2AF65 to Respective G-codes and a Poly-Uridine Tract Collaborate in the N50-5'ss Selection of the REST N Exon in H69 Cells
Source: PLoS One. 2012 Jul 5;7(7):e40315. doi: 10.1371/journal.pone.0040315 (PMC3390395; doi:10.1371/journal.pone.0040315)

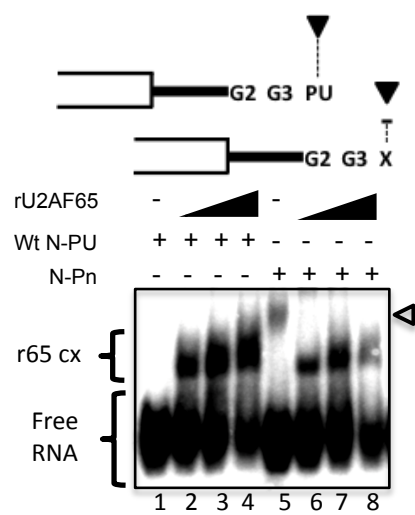

Supplement: Figure S2 — The N-PU is a bona fide poly-Uridine tract recognized by U2AF65 from H69 cells. The wild type N-PU (lanes 1–4) or mutant N-Pn (lanes 5–8) RNAs (shown at the top) were used in EMSA experiments with 2 µg (lanes 2 and 6), 4 µg (lanes 3 and 7) or 6 µg (lanes 4 and 8) of rU2AF65; free probes were run in lanes 1 and 5, respectively. The two remaining uridines in the mutant RNA (UUUUUU to GAUAUC) account for the residual (roughly 20%) rU2AF65 complexes (r65 cx), suggesting that the N-PU is necessary for U2AF65 recruiting to the N exon alternative 5'ss. An empty arrowhead indicates a structured form of the mutant probe. (PDF) [file pone.0040315.s002.pdf]

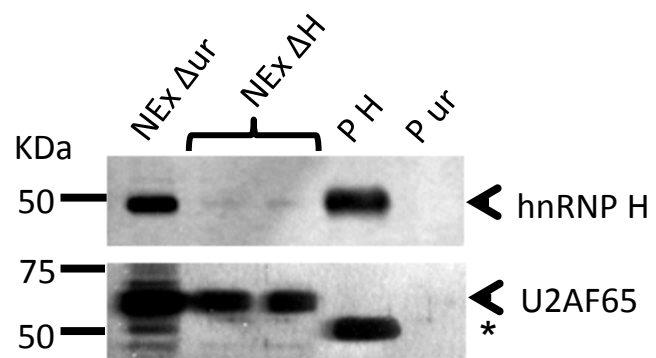

Supplement: Figure S3 — hnRNP H immunodepletion. NEx from H69 cells were depleted using antibodies against an unrelated antigen (Δur) or against hnRNP H (ΔH). hnRNP H antibodies were used to probe western blots after immunodepletion. The hnRNP H (P H) and unrelated proteins (P ur) bound to the pellets after immunodepletions were analyzed also. To control the hnRNP H depletion, the same membrane was stripped and incubated with antibodies against U2AF65 (lower panel). An asterisk indicates residual hnRNP H after stripping. (PDF) [file pone.0040315.s003.pdf]

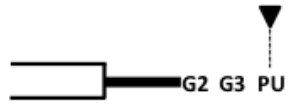

|                 |   |   |   |   |   |   |   |   |
|-----------------|---|---|---|---|---|---|---|---|
| H69 NEx         | - | + | - | - | - | - | - | - |
| $\Delta$ U2AF65 | - | - | + | - | - | - | - | - |
| $\Delta$ unrel  | - | - | - | + | - | - | - | - |
| MRC5 NEx        | - | - | - | - | - | + | + | - |
| rU2AF65         | - | - | - | - | - | - | + | + |

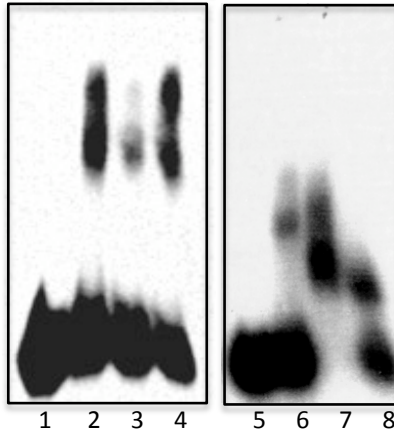

Supplement: Figure S4 — U2AF65 is necessary for complex recruiting at the N50-5'ss. The wild type probe (shown at the top; lanes 1 and 5) was used in EMSA with NEx from H69 cells (lane 2), or with H69 NEx depleted of U2AF65 (ΔU2AF65; lane 3) or depleted of an unrelated antigen (Δunrel; lane 4). Complex formation was reduced with the ΔU2AF65 NEx. Reciprocally, the probe was incubated with NEx from MRC5 cells alone (lane 6) or complemented with rU2AF65 (lane 7). rU2AF65 was required for shifting the mobility of the complex and the remaining probe, in a different manner than rU2AF65 alone (lane 8). (PDF) [file pone.0040315.s004.pdf]

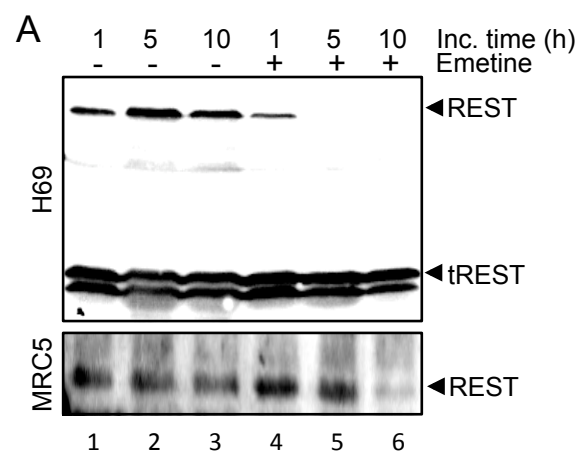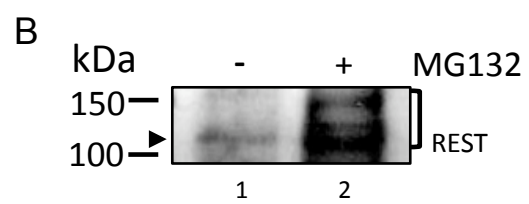

Supplement: Figure S6 — cREST is readily degraded in H69 cells. (A) Western blots from emetine-treated (+; 100 μg/mL) or untreated (-) H69 (upper panel) and MRC5 cells (lower panel). Incubation times were 1, 5 and 10 h. Canonical (cREST) and truncated (tREST) isoforms are indicated. (B) Nuclear extracts from H69 cells treated (+) or not (-; 0.2% DMSO only) with the proteasome inhibitor MG132 at 10 μM final concentration during 4 h were probed with anti-REST antibody. REST (arrowhead in lane 1) and ubiquitinated-REST (brackets in lane 2) are shown. (PDF) [file pone.0040315.s006.pdf]

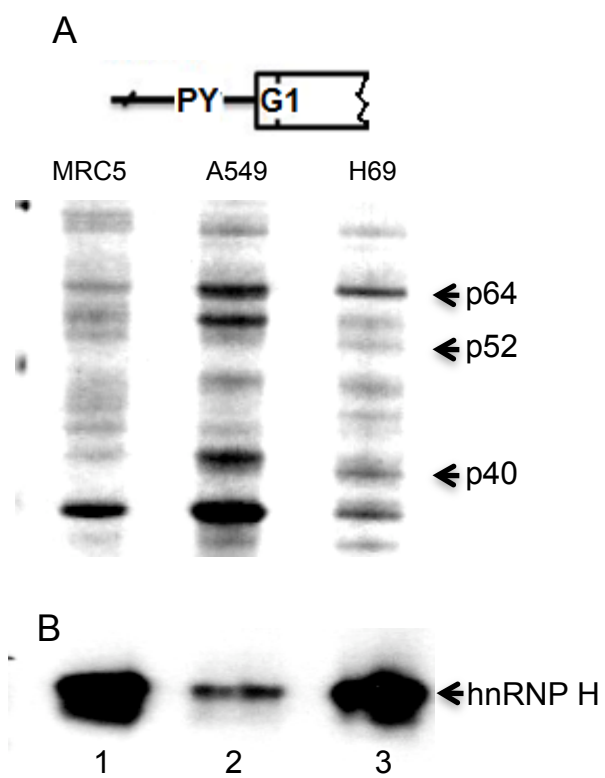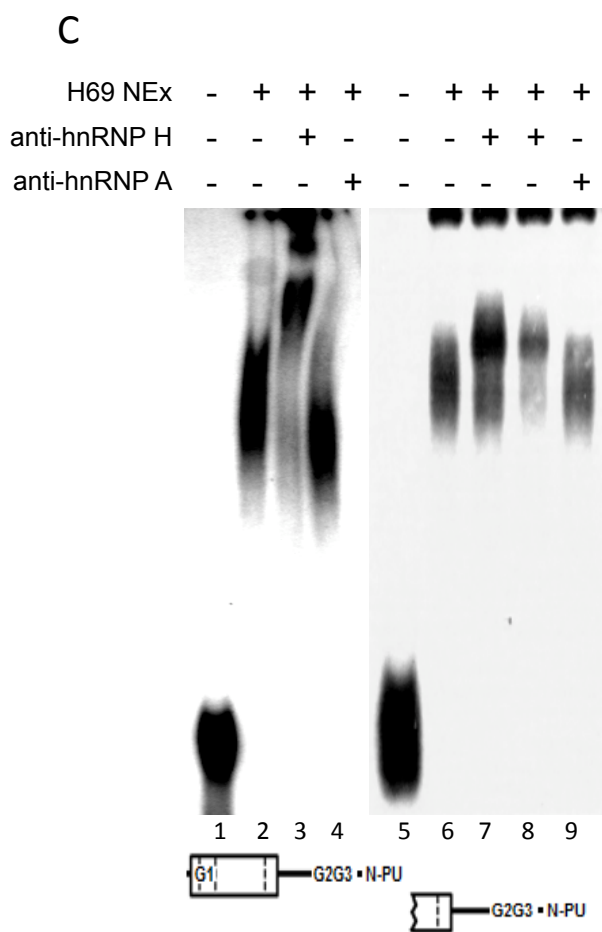

Supplement: Figure S7 — hnRNP H from H69 cells is able to bind to all G codes. (A) Twenty fmole of a 32P-labeled RNA probe (shown on top) containing part of the N4-5ss only, the 3'ss, the Py and part of the upstream intron were incubated with 5 µg of each NEx from MRC5, A549 (other SCLC cell line) or H69 cells. The EMSA complexes were resolved in a 4% native polyacrylamide gel. Proteins from 20 complexes were purified and visualized in silver stained 10% SDS-polyacrylamide gels. Black arrows show the proteins that were differentially purified from NEx. (B) A sample of the purified complexes was electrophoresed and probed with anti-hnRNP H antibodies by western blot. A strong signal for hnRNP H was detected in MRC5 (lane 1) and H69 (lane 3) cells indicating that hnRNP H is recruited to the G1. A less intense hnRNP H signal was observed in A549 NEx. (C) Two different RNA probes (drawings at the bottom), with or without the G1 element, were labeled (lanes 1 and 5, respectively) and used in EMSA with H69 NEx. Both probes rendered complexes with the NEx (lanes 2 and 6) but that with the G1 element was supershifted more efficiently with the anti-hnRNP H antibodies (lane 3) as compared with the modest supershift obtained with the probe lacking the G1 element (lanes 7 and 8; antibody added before or after the probe). Antibodies against hnRNP A did not supershifted the complexes formed with either probe. (PDF) [file pone.0040315.s007.pdf]

|                       |   |   |   |   |   |   |
|-----------------------|---|---|---|---|---|---|
| NEx c.o.              | - | + | - | - | - | - |
| NEx U1 k.o.           | - | - | + | - | - | - |
| H69 NEx               | - | - | - | + | - | - |
| $\Delta$ U2AF65       | - | - | - | - | + | - |
| $\Delta$ 65 + rU2AF65 | - | - | - | - | - | + |

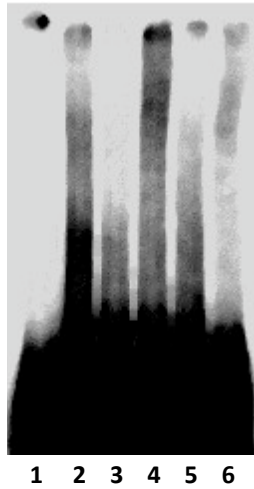

Supplement: Figure S8 — Depletion of U1 snRNA or U2AF65 of H69 NEx inhibits complex recruiting around the N exon. The wild type probe (lane 1) was used in EMSA with NEx from H69 cells (lane 4), or with H69 NEx depleted of U2AF65 (ΔU2AF65; lane 5) or with NEx in which the 5' end of U1 snRNA was degraded by RNase H-mediated digestion with a complementary oligonucleotide (NEx U1 k.o.; lane 3). Complex formation was not diminished when the U1 snRNA was degraded with a scrambled oligonucleotide as a control (NEx c.o.; lane 2) and was partially recovered when the ΔU2AF65 extracts were supplemented with recombinant U2AF65 (Δ65 + rU2AF65; lane 6). (PDF) [file pone.0040315.s008.pdf]
