# Supplementary material for: Binding of hnRNP H and U2AF65 to Respective G-codes and a Poly-Uridine Tract Collaborate in the N50-5'ss Selection of the REST N Exon in H69 Cells
Source: PLoS One. 2012 Jul 5;7(7):e40315. doi: 10.1371/journal.pone.0040315 (PMC3390395; doi:10.1371/journal.pone.0040315)

A

```
         RRAD   CCCAGCCCGGCGGCCTGGGCC
NRSE consensus  TTCAGCACCACGGACAGCGCC
         TMS1   TGGAGGGCAACGGACCGGGGC
```

B

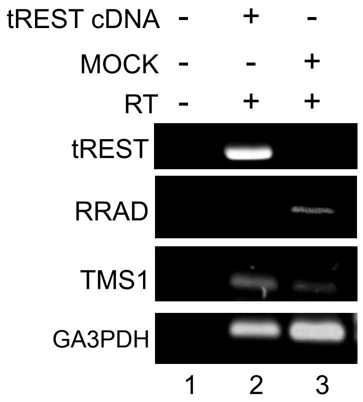

Supplement: Figure S5 — Transfection of the cDNA for tREST into MRC5 cells modifies RRAD and TMS-1 basal expression. (A) Putative binding sites for REST were localized close to the TATA box in the promoters of the human RRAD and TMS-1 genes. (B) RT-PCR showing the altered expression of RRAD and TMS-1 in cells transfected with the tREST cDNA (lane 2), compared to the MOCK transfections (lane 3), and no RT controls (lane 1). The GA3PDH amplicon was used for loading control. (PDF) [file pone.0040315.s005.pdf]
